# Sarcopenia index based on serum creatinine and cystatin C predicts the risk of postoperative complications following hip fracture surgery in older adults

**DOI:** 10.1186/s12877-021-02522-1

**Published:** 2021-10-12

**Authors:** Xiaoyan Chen, Yanjiao Shen, Lisha Hou, Binyu Yang, Birong Dong, Qiukui Hao

**Affiliations:** 1 Zigong Mental Health Center, Zigong, Sichuan Province China; 2 Department of Guideline and Rapid Recommendation, Cochrane China Center, MAGIC China Center, Chinese Evidence-Based Medicine Center, West China Hospital, Sichuan University, Chengdu, Sichuan Province China; 3 National Clinical Research Center for Geriatrics, West China Hospital, Sichuan University, Chengdu, Sichuan Province China; 4 Chengdu Jinxin Mental Diseases Hospital, Chengdu, Sichuan Province China

**Keywords:** Sarcopenia index, Creatinine, Cystatin C, Hip fracture, Older adults, Postoperative complications

## Abstract

**Objective:**

To assess the utility of the preoperative Sarcopenia index (SI) as a predictive marker of the risk of postoperative complications following hip fracture surgery in older adults.

**Study design:**

This observational study enrolled older adults with hip fracture who were hospitalized in the Department of Orthopedics of West China Hospital, Sichuan University, from December 7, 2010 - June 14, 2017, and who underwent hip fracture surgery.

**Primary outcome and measures:**

Clinical data were collected from medical records and serum creatinine and cystatin C were measured before surgery. Outcomes included postoperative complications such as pneumonia, urinary tract infection, respiratory failure, heart failure, and non-grade A healing. Binary logistic regression analyses were used to analyze association between SI and postoperative complications.

**Results:**

A total of 897 patients aged 60 years and over were enrolled in this study (age range: 60 – 100 years), of whom 306(34.1%)were male, and 591(65.9%)were female. Postoperative complications included pneumonia (12%), urinary tract infections (1.8%), respiratory failure (1.5%), heart failure (1.6%), and non-A- grade healing (3.6%). In the patient group that received joint replacements, the incidence of pneumonia was negatively associated with SI values. After adjusting for potential confounding factors, binary logistic regression analyses showed that a higher SI was independently associated with a lower risk of pneumonia after joint replacement surgery (OR:0.39, 95% CI:0.18-0.89, *P*<0.05). However, we did not find statistically significant association between SI and the risk of postoperative complications other than pneumonia among patients with two types of hip fracture surgery.

**Conclusion:**

The SI based on serum creatinine and cystatin C can predict pneumonia rather than other postoperative complications among older patients with hip fracture after joint replacement surgery.

## Background

Hip fracture is common in older adults, and is usually treated via surgical approaches that promote fracture healing and shorten the course of the disease. However, there is a high risk of postoperative complications in hip fracture patients [1–3]; thus, the prevention of such complications is an area of active research. Sarcopenia can lead to poor prognosis and even death after hip fracture surgery [4–6] indicating the importance of preoperative identification of sarcopenia. Sarcopenia is an age-related disorder that results in the loss of skeletal muscle mass and muscle strength, and/or reduced physical performance [7]. Low muscle mass can usually be quantified through imaging approaches such as dual-energy x-ray absorptiometry (DXA), bioelectrical impedance analyses (BIA), magnetic resonance imaging (MRI), or computed tomography (CT). These approaches, however, require specific instruments and are expensive, with MRI and CT scans necessitating the use of a special image-processing system to calculate muscle mass. More reliable, accessible, and cost-effective approaches are thus necessary to better characterize muscle mass loss.

In recent years, the sarcopenia index (SI, serum creatinine [mg/dl]/cystatin C [mg/l] × 100) has been recommended to evaluate skeletal muscle mass [8]. The SI remains largely constant regardless of renal function, and the two indicators are obtained from the serological results of hospitalized patients, which are readily available and convenient. Tetsuka et al. found the SI to be an effective predictor of muscle mass in amyotrophic lateral sclerosis [9], with SI declining as disease severity increased. Kashani et al. [8] enrolled 226 patients in an intensive care unit (ICU) and found the correlation (r) between SI and muscle mass to be 0.62, further showing that SI could predict hospital and 90-day mortality among patients who did not exhibit acute kidney injury at the time of measurement. Barreto et al. also found SI to be independently related to muscle mass. Decreases in SI were also related to frailty and poorer short-term ICU patient clinical outcomes [10]. At present, no studies have explored the predictive role of SI in patients after hip surgery. As such this study sought to evaluate the relationship between SI and postoperative complications after hip fracture surgery in older adults.

## Methods

### Study design and patient recruitment

This retrospective observational study was conducted in West China Hospital, Sichuan University, Chengdu, Sichuan Province, China. This study was conducted in line with the Declaration of Helsinki and was approved by the Ethical Review Committee of West China Hospital of Sichuan University with the committee’s reference number 2017(445) and the registration number is 2018-95. The Ethical Review Committee of West China Hospital of Sichuan University waived the need for informed consent in this study.

Older adults with hip fracture who were hospitalized in the Department of Orthopedics of this hospital from December 7, 2010, to June 14, 2017, and who underwent hip fracture surgery were eligible for study enrollment. In this study, we enrolled patients with internal fixation surgery and joint replacement surgery. Due to the different surgical methods and recovery time of patients in the internal fixation group and the joint replacement group, the incidence of postoperative complications was different. Therefore, we analyzed the association of SI and postoperative complications separately according to the surgical methods. Inclusion criteria: 1) hospitalized patients aged 60 years and over; 2) in cases of repeated analyses of the same patient, only the most recent data were analyzed. Exclusion criteria: 1) patients with both hip fractures and tumors; 2) patients with severe renal dysfunction (eGFR< 15 mL/min/1.73 m^2^); 3) patients with incomplete clinical medical records that precluded measurements of the SI; 4) patients with incomplete surgical records or without information on surgical complications and risks.

### Measures

General indicators such as age, sex, smoking history, disease, serum albumin (ALB), postoperative complications mainly diagnosed according to ICD-10 (pneumonia, urinary tract infection, respiratory failure, heart failure) and clinician's diagnosis of incision recovery (non-A-grade healing) were collected from the hospital database. The normal ALB range is 35-55 g/L, and a level < 35 g/L was considered low [11].

### Primary outcome

Experienced nurses drew fasting (more than 8 h) venous blood in the morning before surgery from every patient. Serum cystatin C concentration (mg/l) and cystatin C were measured in West China Hospital laboratory using standard methods. We calculated the SI using the following formula: serum creatinine value/cystatin C value × 100 [8]. According to the SI values, all participants were separated into two groups (low or high muscle mass). The SI median was used as the cut-off value with low muscle mass defined as lower than the median and high muscle mass as equal to or higher than the median. Outcomes included postoperative complications, such as pneumonia, urinary tract infection, respiratory failure, heart failure, and non-A- grade healing.

### Statistical analysis

Results were analyzed using SPSS 26.0 (IBM Corp, Armonk, NY, USA). Categorical data were reported as numbers (percentages), and Pearson’s chi-square tests were used when comparing baseline characteristics. For continuous variables, we first tested whether they are normally distributed. If they were, they were represented by the mean (standard deviation). Otherwise, use a four-fold interval (IQR). One-way ANOVA was used to detect differences between groups. Binary logistic regression analysis was used to analyze the association between SI and postoperative complications. Two models were used for this regression analysis: model 1 was unadjusted, while model 2 was adjusted for confounder variables, including age, sex, smoking history, drinking history, body mass index (BMI), estimated glomerular filtration rate (eGFR), ALB level, methods of anesthesia, and chronic diseases (hypertension, diabetes, coronary heart disease, chronic obstructive pulmonary disease). We adjusted these variables for we judged that these factors related to the postoperative complications. In this study, *P* < 0.05 was the threshold of significance.

## Results

### Study participant characteristics

A total of 897 patients aged 60 years and above were included in this study (mean age: 76.9, SD: 8.8, range: 60 – 100 years), of whom 306(34.1%)were male, and 591(65.9%)were female. The proportions of patients aged 60-70 years and > 70 years were 24.5% and 75.5%, respectively. In this study, 556 (62%) patients received internal fixation surgery and 341(38%) patients received joint replacement surgery. We divided all participants into two groups according to the type of surgery (the internal fixation group and the joint replacement group. Furthermore, the participants were further divided into two groups according to the median of SI. Low muscle mass was defined as SI<74.29 for all participants, SI<74.34 for participants in internal fixation group, SI<74.16 for participants in joint replacement group. Participants with SI equal to or above these medians were defined as high muscle mass. We observed that the two groups differed significantly in age, sex, ALB level, BMI, eGFR, smoking history, drinking history, and marital status in all participants. There were significant differences between the two SI groups for patients who had received internal fixation in terms of age, sex, ALB level, BMI, eGFR, marital status, and diabetes. In patients that received joint fixation, significant differences in sex, ALB level, eGFR, smoking history, drinking history, and marital status were observed between the two groups (Table 1).

**Table 1:** Baseline characteristics of participants according to the sarcopenia index

Characteristics	Total ***n*** = 897			Internal fixation ***n*** = 556			Joint replacement ***n*** = 341		
	low muscle mass	high muscle mass	p	low muscle mass	high muscle mass	p	low muscle mass	high muscle mass	p
**Age, years, n(%)**			<0.001			0.004			0.053
60-70	87(19.4)	133(29.7)		55(19.7)	84(30.3)		33(19.3)	48(28.2)	
>70	362(80.6)	15(70.3)		224(80.3)	193(69.7)		138(80.7)	122(71.8)	
**Age, years, mean (SD)**	78.3(8.5)	75.5(8.9)		77.9(8.6)	75.3(8.9)		78.8(8.4)	75.9(8.9)	
**Sex, n(%)**			<0.001			<0.001			<0.001
male	95(21.2)	211(47.1)		65(23.3)	124(44.8)		30(17.5)	87(51.2)	
female	354(78.8)	237(52.9)		214(76.7)	153(55.2)		141(82.46)	83(48.8)	
**ALB level, g/l, n(%)**			<0.001			<0.001			0.027
<35 g/l	192(42.8)	124(27.7)		121(43.4)	71(25.6)		72(42.1)	52(30.6)	
≥ 35 g/l	257(57.2)	324(72.3)		158(56.6)	206(74.4)		99(57.9)	118(69.4)	
**BMI, kg/m**^**2**^**, mean (SD)**	21.70(3.13)	22.42(3.37)	0.001	21.68(3.12)	22.48(3.47)	0.005	21.74(3.14)	22.32(3.21)	0.095
**eGFR, mL/min/1.73 m**^**2**^**, mean (SD)**	97.43(31.23)	78.11(25)	<0.001	97.54(31.72)	77.61(24.94)	<0.001	97.25(30.43)	78.8(25.77)	<0.001
**Smoking history,n(%)**			<0.001			0.163			<0.001
no	398(88.6)	358(79.9)		238(85.3)	224(80.9)		161(94.2)	133(78.2)	
yes	51(11.4)	90(20.1)		41(14.7)	53(19.1)		10(5.8)	37(21.8)	
**Drinking history,n(%)**			<0.001			0.413			<0.001
no	414(92.2)	378(84.4)		249(89.3)	241(87)		166(97.1)	136(80)	
yes	35(7.8)	70(15.6)		30(10.7)	36(13)		5(2.9)	34(20)	
**Marital status, n(%)**			<0.001			0.041			<0.001
married	325(72.4)	375(83.7)		211(75.6)	229(82.7)		115(67.3)	145(85.3)	
single or divorced or widowed	124(27.6)	73(16.3)		68(24.4)	48(17.3)		56(32.7)	25(14.7)	
**Methods of anesthesia,n(%)**			0.589			0.509			0.987
local anaesthesia	48(10.7)	53(11.8)		34(12.2)	39(14.1)		14(8.2)	14(8.2)	
general anesthesia	401(89.3)	395(88.2)		245(87.8)	238(85.9)		157(91.8)	156(91.8)	
**Hypertension,n(%)**			0.969			0.91			0.96
no	257(57.2)	257(57.4)		178(63.8)	178(64.3)		79(46.2)	79(46.5)	
yes	192(42.8)	191(42.6)		101(36.2)	99(35.7)		92(53.8)	91(53.5)	
**Diabetes,n(%)**			0.058			0.026			0.977
no	365(81.3)	341(76.1)		231(82.8)	208(75.1)		134(78.4)	133(78.2)	
yes	84(18.7)	107(23.9)		48(17.2)	69(24.9)		37(21.6)	37(21.8)	
**CHD,n(%)**			0.488			0.279			0.986
no	423(94.2)	417(93.1)		270(96.8)	263(94.9)		154(90.06)	153(90)	
yes	26(5.8)	31(6.9)		9(3.2)	14(5.1)		17(9.94)	17(10)	
**COPD, n(%)**			0.643			0.418			0.772
no	377(84)	371(82.8)		232(83.2)	223(80.5)		146(85.4)	147(86.5)	
yes	72(16)	77(17.2)		47(16.8)	54(19.5)		25(14.6)	23(13.5)	

Note:*ALB* serum albumin; *CHD* coronary heart disease; *COPD* chronic obstructive pulmonary disease

### Postoperative complications

In this study, the observed complications included pneumonia, urinary tract infections, respiratory failure, heart failure, and non-A- grade healing which affected 12%, 1.8%, 1.5%, 1.6%, and 3.6%, respectively, of the overall patients. In the joint replacement group, the incidence of pneumonia was significantly associated with higher SI. However, there were no significant differences in the incidence of urinary tract infection, respiratory failure, heart failure, and non-A-grade healing between the low SI and high SI groups in the patients overall or in the internal fixation or joint replacement groups (Table 2).

**Table 2:** Differences in the distribution of postoperative complications between patients with low sarcopenia index and high sarcopenia index

Characteristics	Total ***n*** = 897			Internal fixation ***n*** = 556			Joint replacement ***n*** = 341		
	SI<74.29	SI ≥ 74.29	p	SI<74.34	SI ≥ 74.34	p	SI<74.16	SI ≥ 74.16	p
**Pneumonia,n(%)**			0.223			0.873			0.025
no	389(86.6)	400(89.3)		244(87.5)	241(87)		146(85.4)	158(92.9)	
yes	60(13.4)	48(10.7)		35(12.5)	36(13)		25(14.6)	12(7.1)	
**Urinary tract infection,n(%)**			0.617			0.991			0.414
no	440(98)	441(98.4)		274(98.2)	272(98.2)		167(97.7)	168(98.8)	
yes	9(2)	7(1.6)		5(1.8)	5(1.8)		4(2.3)	2(1.2)	
**Respiratory failure,n(%)**			0.777			0.992			0.657
no	443(98.7)	441(98.4)		275(98.6)	273(98.6)		168(98.3)	168(98.8)	
yes	6(1.3)	7(1.6)		4(1.4)	4(1.4)		3(1.7)	2(1.2)	
**Heart failure,n(%)**			0.279			0.697			0.249
no	444(98.9)	439(98)		276(98.9)	273(98.6)		169(98.8)	165(97.1)	
yes	5(1.1)	9(2)		3(1.1)	4(1.4)		2(1.2)	5(2.9)	
**Non-a-gradehealing,n(%)**			0.995			0.411			0.101
no	433(96.4)	432(96.4)		268(96.1)	262(94.6)		166(97.08)	169(99.4)	
yes	16(3.6)	16(3.6)		11(3.9)	15(5.4)		5(2.92)	1(0.6)	

There were no significant differences in the correlations between SI and the risk of urinary tract infection, respiratory failure, heart failure, or poor healing in the overall patients or those who had received either internal fixation or joint replacement. In the joint replacement group, model 1 showed that a higher SI was associated with the risk of pneumonia after hip fracture surgery. After adjustment for potential confounding factors, model 2 showed that a higher SI was independently associated with a lower risk of pneumonia after hip fracture surgery in the joint replacement group (odds ratio (OR): 0.39, 95% confidence interval (CI): 0.18-0.89). (Table 3).

**Table 3:** Associations between sarcopenia index and postoperative complications

Adverse reactions	Total		Internal fixation		Joint replacement	
	Model 1	Model 2	Model 1	Model 2	Model 1	Model 2
**Pneumonia**						
low muscle mass (ref)	1	1	1	1	1	1
high muscle mass	0.78(0.52-1.17)	0.75(0.48-1.18)	1.04(0.63-1.71)	1.02(0.61-1.86)	0.44(0.22-0.92)*	0.39(0.18-0.89)*
**Urinary tract infection**						
low muscle mass (ref)	1	1	1	1	1	1
High muscle mass	0.78(0.29-2.10)	1.04(0.35-3.03)	1.01(0.29-3.52)	NA	0.50(0.1-2.8)	NA
**Respiratory failure**						
low muscle mass (ref)	1	1	1	1	1	1
high muscle mass	1.17(0.39-3.52)	1.19(0.35-4.04)	1.01(0.25-4.07)	NA	0.67(0.11-4.04)	NA
**Heart failure**						
low muscle mass (ref)	1	1	1	1	1	1
high muscle mass	1.82(0.61-5.48)	2.31(0.64-8.33)	1.35(0.3-6.08)	1.10(0.16-7.51)	2.56(0.49-13.38)	NA
**Non-a-grade healing**						
low muscle mass (ref)	1	1	1	1	1	1
high muscle mass	1.00(0.5-2.03)	1.26(0.58-2.72)	1.40(0.63-3.09)	1.71(0.72-4.04)	0.2(0.02-1.7)	NA

Model 1: non-adjusted model.

Model 2: adjustment for age, sex, smoking history, drinking history, BMI, eGFR ALB level, methods of anesthesia, and presence of chronic diseases (hypertension, diabetes, coronary heart disease, chronic obstructive pulmonary disease).

*NA*: unable to analyze due to few postoperative complication events.

*: *p*<0.05

## Discussion

In this study, we found a higher SI was independently associated with a lower risk of pneumonia after joint replacement surgery. This is the first study to examine the role of SI in predicting postoperative complications among patients who underwent hip fracture surgery. In addition, we reported stratified results according to type of hip fracture surgery.

The rate of postoperative pneumonia was similar to that reported in previous studies [12–14]. Previous studies have shown that postoperative pneumonia is an important cause of death after fracture surgery. Lawrence et.al. [15] found that 14% of older hip fracture patients with pulmonary infections died within 30 days, while for those non-pulmonary infections, the mortality rate was only 1.7% within 30 days. Another study determined that the risk of death was 7.36 times higher in patients with pulmonary infections after hip fracture relative to patients without pulmonary infection [16]. Thus, clinicians should focus on pneumonia in these patients in an effort to reduce mortality rates. Respiratory muscle strength can regulate coughing effectively to clear the airway [17] and pneumonia is more likely to occur if the muscle mass is reduced. A previous study showed that patients with sarcopenia have a higher risk of pneumonia [18]. We found that the SI can predict the risk of pneumonia in this patient sample, that is, a higher SI was independently associated with a lower risk of pneumonia after hip fracture surgery in the joint replacement group. Therefore, we believe that the SI is an effective surrogate for sarcopenia in predicting postoperative complications.

However, our results cannot be generalized to the patients with internal fixation. The possible reason is that there are more factors influencing the relationship between SI and pneumonia in the internal fixation group than in the joint replacement group. These factors including but are not limited to insufficient physical activity. Compared with patients undergoing joint replacement, patients undergoing internal fixation surgery spend more time in bed with analgesia and pain. Postoperative pneumonia is usually associated with prolonged bed rest, sedation and pain. Since patients are immobile for a long time after surgery, this often leads to insufficient lung expansion, which in turn leads to atelectasis, making it difficult to clear lung secretions, and the presence of sedation or pain also makes it difficult to clear lung secretions[19]. But we still need a larger sample to confirm our findings.

A systematic review and meta-analysis has shown that sarcopenia and heart failure often coexist, with the prevalence of sarcopenia in heart failure patients ranging from 10 to 69%. Both sarcopenia and heart failure appear to have similar pathogenetic pathways [20]. However, previous studies have focused on chronic heart failure. In the present study, we did not find an association between SI and heart failure after hip fracture in older adults. The possible reason is that postoperative heart failure in our study is relatively acute, largely related to surgical stress, inappropriate fluid administration, blood loss, transfusion, or the preoperative discontinuation of diuretics [19].

Lieffers et al. reported that muscle mass loss could predict the postoperative incidence of urinary tract infection [21]. However, we detected no correlation between SI and urinary tract infection rates. This may be because the incidence of postoperative urinary tract infections in our study was very low, to the extent that the present study is underpowered.

Impaired wound healing is a common cause of wound-related complications. Decreases in skeletal muscle mass have been shown to be correlated with a poorer prognosis in surgical patients, resulting in increased rates of postoperative complications, including infections and poor wound healing [22]. Achim et al. [23] retrospectively reviewed the medical charts of 70 patients with squamous cell carcinoma (SCC) and found muscle mass loss to be an independent negative prognostic indicator associated with the development of wound complications after total laryngectomy. As such, they suggested that there may be value in measuring loss of muscle mass in patients prior to surgery, given that this may be a modifiable risk factor. In our study, 3.6% of patients exhibited non-grade A healing although there was no correlation between SI and non-grade A healing. This may be because we did not conduct a detailed classification of wound healing, instead of separating patients into grade A healing and non-grade A healing status cohorts. Our sample size was also limited, and we will need to expand these analyses in the future.

There are multiple limitations to this study. First, design is one of the main limitations of research. This study was carried out in a single institution with a limited sample size and was a retrospective study, which resulted in inevitable selection bias and limited its external validity. Second, we could not assess the actual residual muscle mass in these patients using the DXA or BIA approaches due to a lack of specific equipment. As such, the relationship between residual muscle mass and SI in hip fracture patients was not studied in this paper. Third, the number of events for some postoperative complications and the sample size were small. The conclusion of the present study needs to be confirmed by studies with larger sample sizes.

## Conclusion

The sarcopenia index based on serum creatinine and cystatin C can predict pneumonia rather than other postoperative complications among older patients after hip fracture surgery.

## Data Availability

The datasets used and/or analyzed during the current study available from the corresponding author on reasonable request.

## Abbreviations

SI: Sarcopenia index
DXA: Dual-energy x-ray absorptiometry
BIA: Bioelectrical impedance analyses
MRI: Magnetic resonance imaging
CT: Computed tomography
ICU: Intensive care unit
ALB: Serum albumi
SCC: Squamous cell carcinoma
OR: Odd ratio
CI: Confidence interval
